# Multiple Cavernous Malformation Syndrome in an Infant: A Case Report

**DOI:** 10.7759/cureus.63750

**Published:** 2024-07-03

**Authors:** Maria A Castellaro, Lucila Rodriguez Camarda, Marcela Iragorri

**Affiliations:** 1 Pediatrics, BronxCare Health System, New York, USA; 2 Pediatrics, Hospital of Pediatrics S.A.M.I.C. Prof. Dr. Juan P. Garrahan, Buenos Aires, ARG

**Keywords:** familial cavernous malformations, angioma, cerebral cavernous, syndrome, cavernous malformation

## Abstract

Cerebral cavernomas belong to a group of vascular lesions characterized by varying structural properties and presentations. Clinical manifestations vary among patients and are particularly influenced by age, posing challenges in diagnosis and treatment. Here, we present a pediatric case of a cerebral cavernoma, which is very rare. We further aim to emphasize the importance of a good physical examination.

## Introduction

Cerebral cavernomas (CCMs) are rare vascular malformations that affect 0.4-0.8% of the population and represent 10-20% of cerebral vascular lesions; children themselves make up 25% of affected individuals. Cerebral vascular malformations, specifically, are a group of vascular lesions that can vary in structural properties and hemodynamic flow. These encompass the group of malformations that include aneurysms, Moya Moya disease, arteriovenous malformations, venous anomalies, and cerebral cavernous malformations. They can range in size from a couple of millimeters to several centimeters in width, and their location can vary from patient to patient. Pertinent to this case report, cerebral cavernous malformations are vascular spaces of different sizes and characterized on a single layer of endothelin with no distinct features of arteries or veins [1]. These malformations, in particular, are characterized by their low hemodynamic flow. Important to point out are the mixed vascular malformations, which are the most common form of cerebral cavernous malformation (CCM) that occurs in association with a developmental venous anomaly [2-4].

These malformations can present as single or multiple lesions, with the latter generally associated with a hereditary pattern (sporadic/familial) [1]. The hereditary or familiar pattern represents 20 % of all cavernomas in patients and, as mentioned previously, most commonly occur in multiples and are inherited in an autosomal dominant pattern. Various genetic studies conducted have found a correlation between genetic mutations and a hereditary pattern involving mutation of the following three genes: CCM1, CCM2, and CCM3 [2,4-6].

CCMs have a global incidence of <1%. Although patients with CCM are generally asymptomatic and have no focal physical examination findings, they may present with seizures, focal neurological deficits, or headaches. Symptoms are caused most often by mass effect caused from a bleeding; however, research has found that specific symptoms that arise in non-bleeding cavernomas are associated with location in the brainstem. Research shows that the annual risk of bleeding is 3%; however, recurrence of bleed increases by 20% per year once a cavernoma bleeds for the first time [2,7]. This risk is even greater for brainstem cavernomas. The presence of symptoms has also been linked to location of cavernomas within the brain - those lesions located in the supratentorial region, usually present with seizures, whereas lesions in the infratentorial regions most often present with focal neurological deficits.

Most cases of CCM present between the second and fifth decades of life either incidentally or due to symptoms. Only one-third of patients are diagnosed at pediatric ages. It is important to note that these lesions, although sometimes associated with genetic mutations, can appear sporadically and regress on their own. The gold standard diagnostic modality is magnetic resonance imaging (MRI). A more conservative approach is recommended in asymptomatic patients; however, there are no set guidelines that determine the frequency of imaging for follow-up.

## Case presentation

A five-month-old preterm infant, born at 35 weeks gestation due to maternal gestation hypertension, presented to the outpatient clinic with symptoms of bronchiolitis. Incidentally, the physical examination revealed a wide anterior fontanelle and persistence of the posterior fontanelle, with head circumference at the upper limit of normal. The patient, however, presented with neurodevelopment consistent with age and a normal neurological physical examination. There was no other positive past medical history. Family history was negative for the father, mother, and sibling; however, the mother was illiterate and living in a low socioeconomic situation.

**Figure 1:**
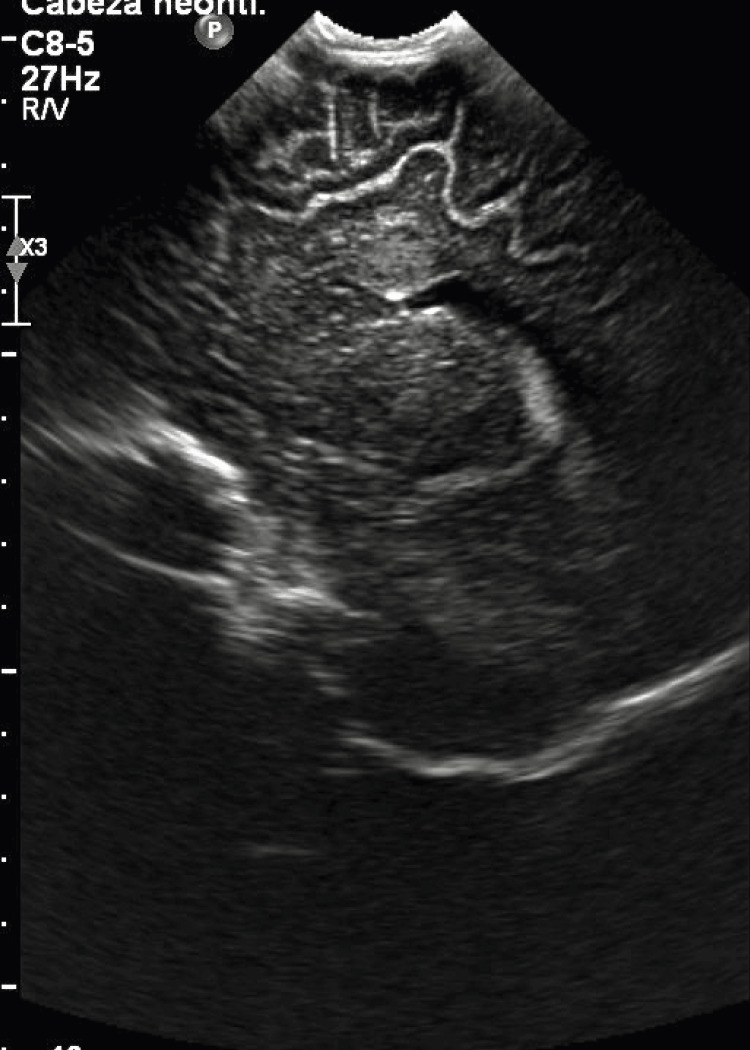
Image of the brain ultrasound showing a rounded image at the level of the roof of the right lateral ventricle of a five-month-old infant.

**Figure 2:**
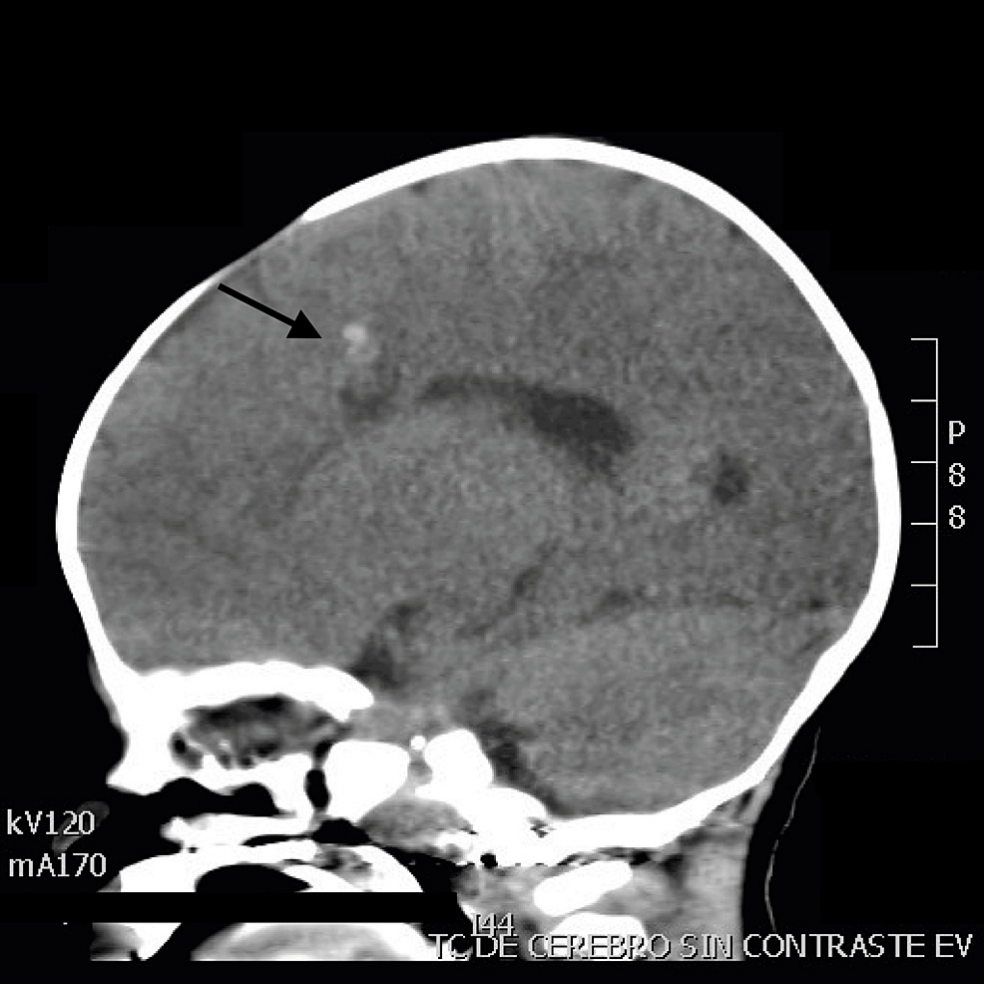
Sagittal plane of CNS CT without contrast. The patient’s largest cavernous malformation is shown in the roof of the left ventricle (black arrows). CNS, central nervous system; CT, computed tomography

**Figure 3:**
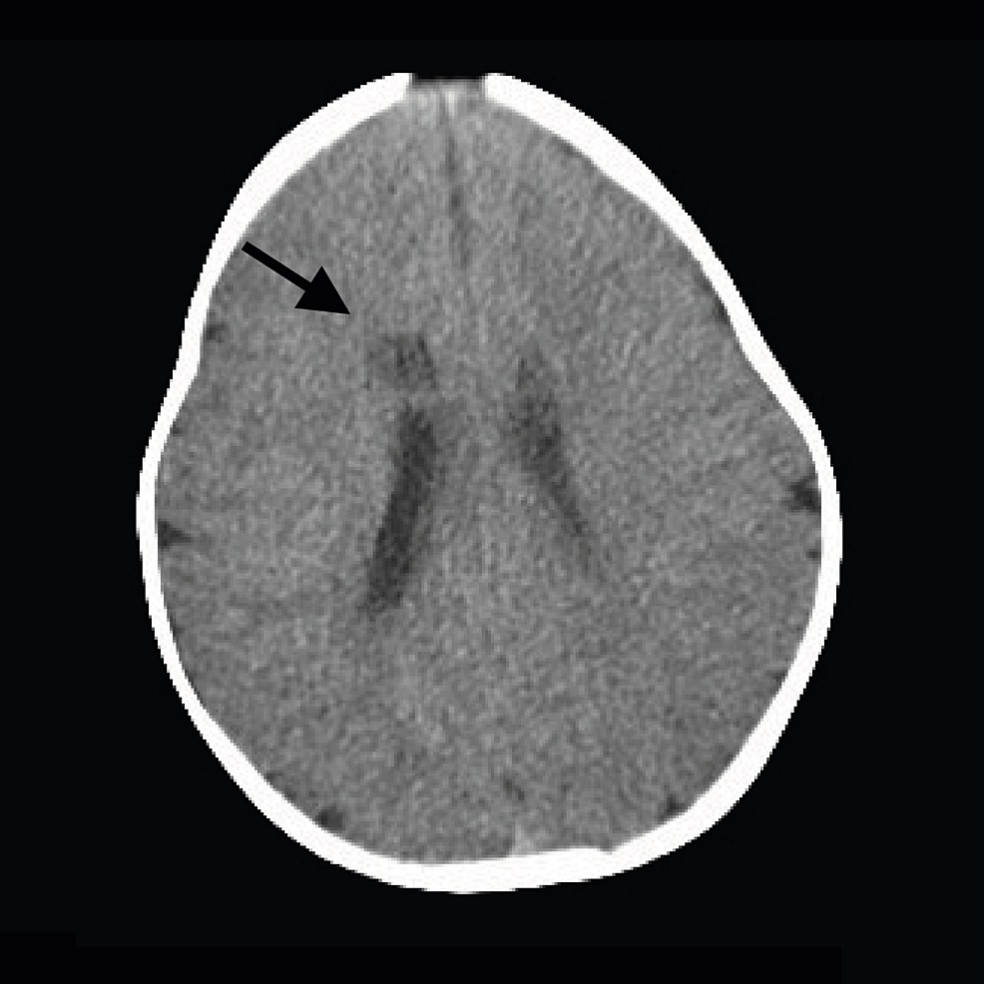
Axial plane of head CT without contrast. Cavernous malformation can be seen (black arrow).

Based on the physical examination findings, the following studies were requested. Brain ultrasound showed a circular structure with homogenous nature measuring ~1.2 cm x 1 cm size with no flow noted on color doppler examination localized to the roof of the right lateral ventricle. No ventricular dilations or midline deviations were detected (Figure 1). Further studies were conducted with the purpose to rule out malignancy versus infectious cause. Computed tomography (CT) of the central nervous system (CNS) without contrast demonstrated a oval, hypodense image, with well-defined borders, measuring approximately 1.2 to 1 cm in diameter, located at the level of the right frontal white matter, adjacent to the roof of the lateral ventricle ipsilateral to which it imprints, which presents adjacent to the upper pole a spontaneously hyperdense area with calcification measuring approximately 6 x 5 mm. A small left frontal cortical calcification, 35 mm in diameter, was also visualized (Figures 2, 3).

**Figure 4:**
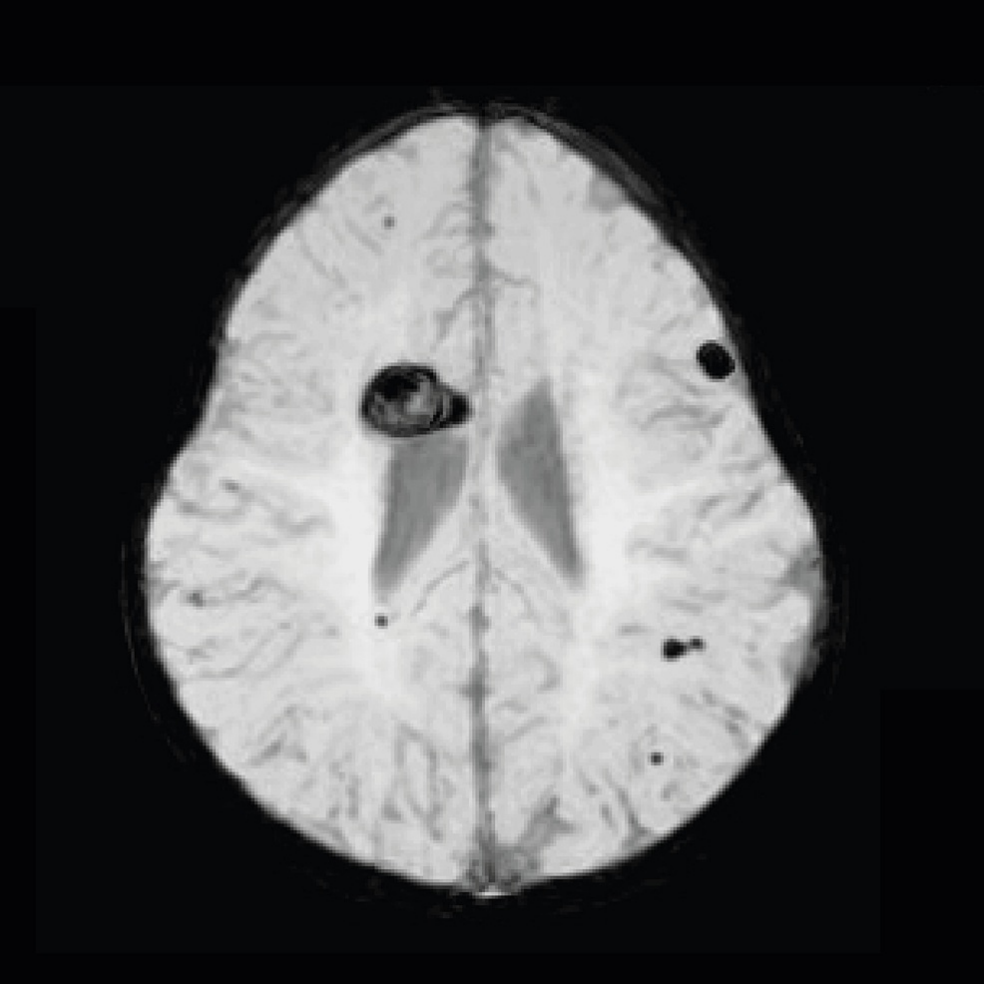
Susceptibility-weighted image from brain MRI. Multiple cavernous malformations are seen in our patient. Largest mass is in the roof of the left ventricle. Cavernous malformation is also seen in the brain, distributed in both hemispheres of the five-month-old patient.

**Figure 5:**
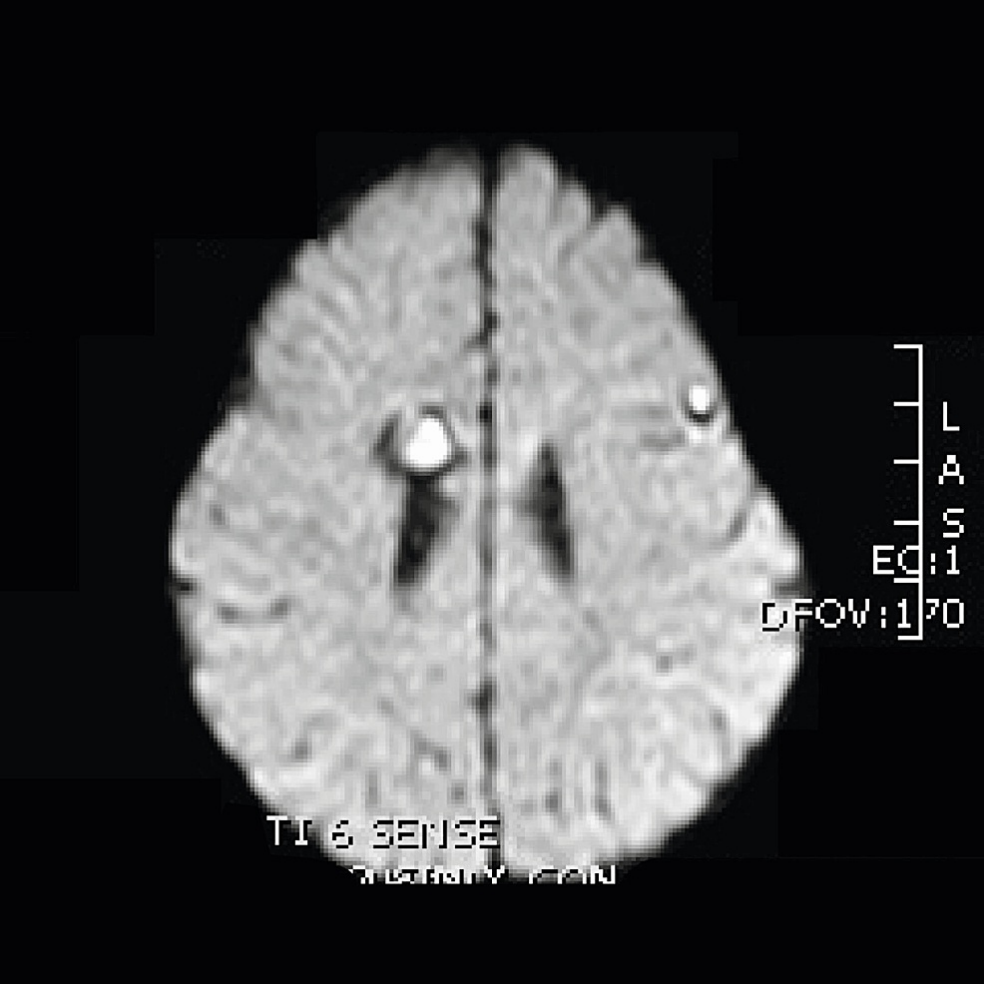
Diffusion-weighted image from head MRI of the five-month-old infant.

CNS MRI showed multiple hypointense images on T2 and weighted sequence, in addition to T1 in the right subcortical and deep temporal parenchyma and bifrontal, with the largest of these measuring 18 mm in maximum diameter and with hyperintense content on T1 and T2 in the right anterior cingulate gyrus. Injection of intravenous contrast did not enhance abnormal structures (Figures 4, 5). Intracranial arterial and venous angio-MRI showed no alterations in caliber or flow signal.

**Figure 6:**
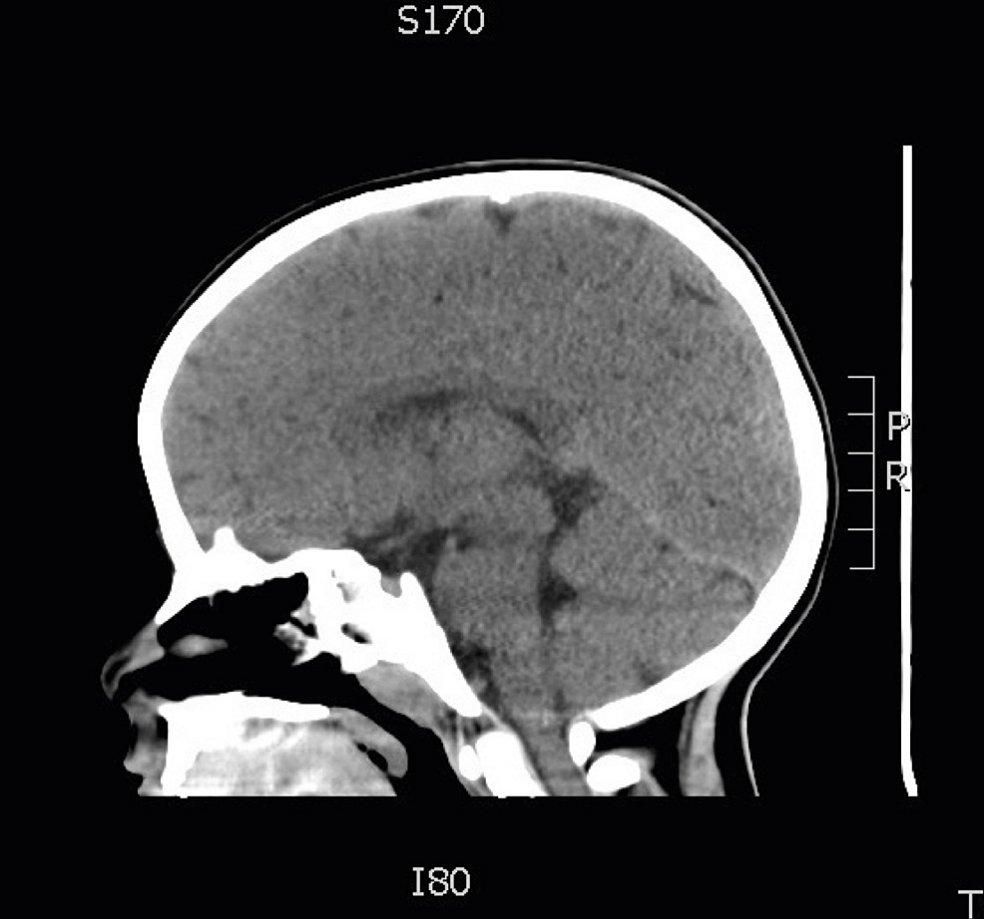
Sagittal CT performed at four years of age showing no obvious lesions in brain parenchyma.

At this time, Neurosurgery was consulted, and a decision was made for close periodic follow-up as no surgery was justified at the time. The patient remained asymptomatic throughout care. The patient was followed up sporadically over the next five years. The last CT was performed at age 4, with no abnormal findings in the parenchyma (Figure 6). MRI was requested but not performed due to loss of the patient to follow-up. Records indicate no seizure activity or bleeding to date.

## Discussion

This case illustrates a rare presentation of pediatric CCM, which presented with multiple lesions and was diagnosed at a routine physical examination. CCMs or cavernous angiomas are congenital cerebral vascular malformations, consisting of multilobed cavities of different sizes, well delineated, which contain blood in different evolutionary stages. Its walls are made up of an endothelium of connective tissue without elastic or muscular fibers and without intervening nervous tissue [1]. These cavernomas are the only of its kinds that exclusively affect the venous system [1-3].

CCMs are rare, especially in pediatric age, and the majority remain asymptomatic, as is the case of our patient, being diagnosed as an incidental finding on imaging after a thorough physical examination during a routine follow-up appointment [1,4-6].

In cases where clinical manifestations occur, literature reports these occurring between the second and fifth decades of life, highlighting the importance of close follow-up [1,4]. The most common symptoms observed are seizures, focal neurological deficits, and headaches. If seizures are present and correlated by EEG and MRI with the location of the lesion, the patient might benefit from surgical resection [3].

The location of these cavernomas is 75-80% supratentorial, as in our case, and they can be single or multiple [3]. Multiple cavernous malformation syndrome is more frequently associated with familial inheritance. The suspicions may be confirmed by genetic testing since there are three genes known to cause mutations: KRIT-1 (CCM-1), CCM-2, and PDCD-10 (CCM-3) [1,4,6].

Although the patient did not have a family history, it would be prudent to pursue genetic testing in such cases.

MRI is the imaging modality of choice for diagnosis and follow-up, especially in gradient echo sequence or even susceptibility-weighted imaging (SWI) since it is more sensitive for its detection. Its appearance is pathognomonic on T2-weighted sequences as a well-defined lobulated mass with a heterogeneous central area (hemorrhages in different evolutionary stages) and a peripheral ring of lower signal intensity (hemosiderin deposits), which gives the lesion a “popcorn” appearance. As these lesions are low flow in nature, angiographic evaluation tends to be negative and not usually recommended. The importance of identification and follow-up is to evaluate the risk of bleeding relapse [4].

A conservative approach is recommended with clinical and imaging controls. Surgical treatment is reserved for symptomatic cases, if they are accessible through this route and can be completely removed, since partial excisions increase the risk of rebleeding [1,6]. Patients presenting with bleeding or hemorrhages associated with CCM have a heightened risk of subsequent hemorrhagic events, often clustered within the first few years after the initial occurrence. This temporal clustering underscores the importance of close monitoring and early intervention strategies to mitigate the risk of re-bleeding and associated neurological complications.

CCMs are rare, especially in pediatric age, and the majority remain asymptomatic, as is the case of our patient, being diagnosed as an incidental finding on imaging [1]. Prognosis of neurodevelopment and cognitive development was unclear at this time, with no strong evidence to support different approaches.

## Conclusions

We have presented a case of a pediatric patient with multiple cavernous malformation syndrome, a rare and infrequent pathology encountered in clinical practice. It is important to know its epidemiological, clinical, and prognostic factors for appropriate monitoring, treatment, and advice of these patients. Seizures are the most frequent and common clinical presentation, when symptomatic. There is no association in the literature that suggests a normal physical examination in a previously healthy patient with no focal neurological deficits with multiple cavernous malformation syndrome, indicating that clinicians should not rule in cavernous malformation syndrome in healthy patient. There is no strong evidence to support the use of anticonvulsants for seizure prophylaxis. However, the current recommendations are for screening for other cutaneous and retinal vascular lesions, genetic testing, and regular follow-up for patients with serial MRI in those with a positive family history. It is important to acknowledge that if bleeding is present at diagnosis, there is an increased subsequent risk of hemorrhagic events. This temporal clustering underscores the importance of close monitoring and early intervention strategies to mitigate the risk of re-bleeding and associated neurological complications. Caution is recommended for the use of NSAIDs, heparin, or warfarin due to the concern for increased risk of bleeding.
